# Holocene variations in peatland methane cycling associated with the Asian summer monsoon system

**DOI:** 10.1038/ncomms5631

**Published:** 2014-08-19

**Authors:** Yanhong Zheng, Joy S. Singarayer, Peng Cheng, Xuefeng Yu, Zhao Liu, Paul J. Valdes, Richard D. Pancost

**Affiliations:** 1 Organic Geochemistry Unit, Cabot Institute and School of Chemistry, University of Bristol, Cantock’s Close, Bristol BS8 1TS, UK; 2 State Key Laboratory of Continental Dynamics, Department of Geology, Northwest University, Xi’an 710069, China; 3 Department of Meteorology, University of Reading, Earley Gate, PO Box 243, Reading, RG6 6BB, UK; 4 State Key Laboratory of Loess and Quaternary Geology, Institute of Earth Environment, Chinese Academy of Sciences, Xi’an 710075, China; 5 Bristol Research Initiative for the Dynamic Global Environment, Cabot Institute and School of Geographical Sciences, University of Bristol, University Road, Bristol BS8 1SS, UK

## Abstract

Atmospheric methane concentrations decreased during the early to middle Holocene; however, the governing mechanisms remain controversial. Although it has been suggested that the mid-Holocene minimum methane emissions are associated with hydrological change, direct evidence is lacking. Here we report a new independent approach, linking hydrological change in peat sediments from the Tibetan Plateau to changes in archaeal diether concentrations and diploptene δ^13^C values as tracers for methanogenesis and methanotrophy, respectively. A minimum in inferred methanogenesis occurred during the mid-Holocene, which, locally, corresponds with the driest conditions of the Holocene, reflecting a minimum in Asian monsoon precipitation. The close coupling between precipitation and methanogenesis is validated by climate simulations, which also suggest a regionally widespread impact. Importantly, the minimum in methanogenesis is associated with a maximum in methanotrophy. Therefore, methane emissions in the Tibetan Plateau region were apparently lower during the mid-Holocene and partially controlled by interactions of large-scale atmospheric circulation.

## Abstract (web summary)

Although it has been widely suggested that the mid-Holocene minimum methane emissions are associated with hydrological change, direct evidence is missing. Here, the authors present evidence from the Tibetan Plateau using tracers of methanogenesis and methanotrophy, in combination with climate simulations.

## Graphical abstract

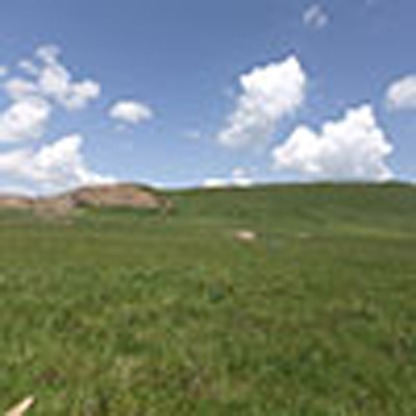

Atmospheric methane (CH_4_) concentrations reached a maximum in the early Holocene (~10 ka BP) of ~700 p.p.b.v. and subsequently decreased to a minimum of ~550 p.p.b.v. during the mid-Holocene from 6 to 4 ka. However, the cause of the minimum remains controversial. Proposed mechanisms invoke the loss of the subtropical methane source^1^, reduced emissions of low-latitude northern hemisphere monsoonal regions^2^, drying of tropical wetlands^3^ and a decreased extent of both northern and tropical peatlands^4^. These changes could be related to the behaviour of the intertropical convergence zone via its influence on monsoon systems (ITCZ^5^,^6^). Insolation-driven summer monsoon variations have been invoked to explain glacial–interglacial variations in atmospheric CH_4_ concentrations^7^, and multiple lines of evidence also demonstrate a gradual decrease in Northern Hemisphere monsoon intensity over the Holocene^5^,^6^,^8^. This appears to have caused drying of wetlands^4^, and could have caused decreased methane emissions, through the Holocene^2^; however, direct evidence is missing. To better understand CH_4_ variations during the Holocene, and to understand how the Asian Monsoon (AM) could have influenced wetland methane dynamics, direct investigations of wetland deposits are required.

We have examined the impact of monsoon-driven hydrological change on methane cycling in Tibetan peats (Fig. 1c) using biomarker proxies for methanogen biomass^9^ and methanotrophy^10^. Although wetland methane flux is governed by a range of factors such as water-table position, temperature, substrate quality and vegetation, the putative precipitation-based mechanisms described above^1^,^2^ bring about lower atmospheric CH_4_ in the mid-Holocene via decreased methanogenesis. Consequently, records of methanogen biomass could serve as a direct test of these hypotheses. Methanogenesis, the biogenic production of CH_4_ mediated by Archaea, is widespread in natural peatlands. Examination of microbially mediated CH_4_ biogeochemical processes in ancient sediments is challenging; however, archaeal diether biomarkers have proved to be useful in studying methanogenic processes in peatlands^9^,^11^. We compare archaeol and hydroxyarchaeol concentrations in a Tibetan Plateau peat to local evidence for bog wetness and regional records of precipitation to evaluate the linkages between the AM and methanogenesis and directly test proposed models for Holocene methane production. We complement our interpretation by evaluating changes in methanotrophy using bacterially derived hopanoid δ^13^C values. Our results indicate that variations in precipitation associated with the Asian summer monsoon (ASM) govern both methanogenesis and methanotrophy in Tibetan peats.

## Results

### Holocene variations of ASM precipitation

**Figure 1:**
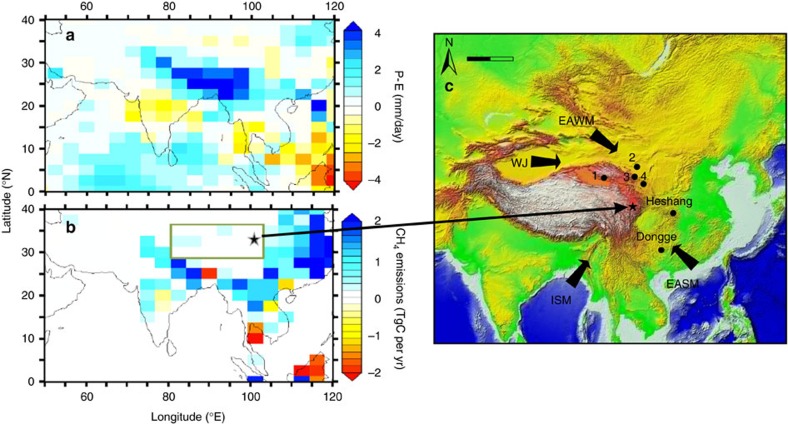
Site location with modelled spatial patterns of precipitation–evaporation and methane emission changes for the Holocene. Anomalies in precipitation–evaporation (P–E) (**a**) and annual methane emissions (**b**) for early Holocene maximum minus late mid-Holocene minimum values (derived using approaches described in ref. 2). The black star shows the site location and the rectangle shows the area for time series averaging. (**c**) Site location and atmospheric circulation (Scale bar, 1,000 km), including sites where other climate records have been developed: 1—Hurleg lake; 2—Sanjiaocheng; 3—Gulang; 4—Jingyuan. ISM, Indian summer monsoon; EASM, East Asian summer monsoon; EAWM, East Asian winter monsoon; WJ, Westerly jet.

The Hongyuan Peat was collected from the Northeastern Tibetan Plateau (32°46′N, 102°31′E; Fig. 1), and chronology was obtained by AMS ^14^C ages (See Methods). The Tibetan peatlands are the largest in China and the largest high-altitude marsh in the world, comprising 600,000 hectares, a small but significant component of the 530–570 mha of wetlands globally^12^,^13^. They contribute between 0.56 and 1 Tg of methane emission per year^14^,^15^,^16^, ~0.5–1% of the average annual global flux from wetlands (110 Tg). The Hongyuan peatlands are dominated by high-cold sedges, with *Carex muliensis* and *Kobresia humilis* being the two major peat-forming species. *C. muliensis* is one of the most abundant plant remains in Hongyuan peats^17^. Other species include *Polygonum viviparum* and *Chamaesium paradoxum*.

**Figure 2:**
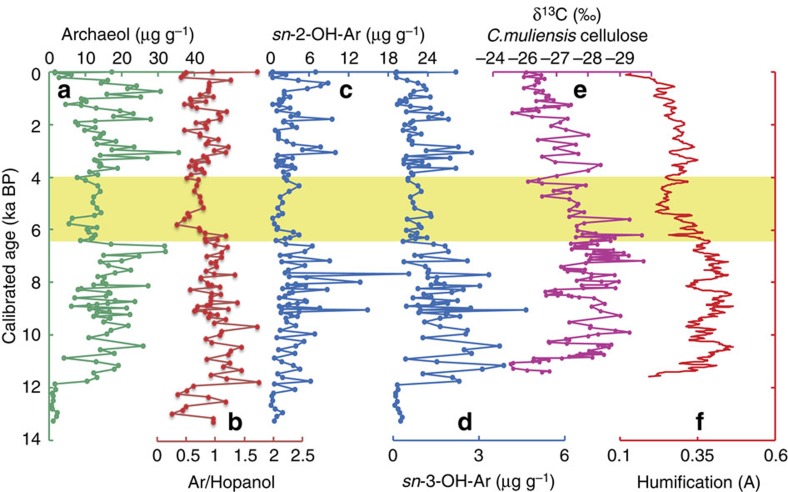
Comparison of microbial lipid and Tibetan Plateau precipitation records. (**a**) Archaeol records. (**b**) The ratio of archaeol to hopanol (Ar/Hopanol). (**c**,**d**) *sn*-2 hydroxyarchaeol (*sn*-2-OH-Ar) and *sn*-3 hydroxyarchaeol (*sn*-3-OH-Ar) concentrations. (**e**,**f**) *Carex muliensis* cellulose δ^13^C values^17^ as well as humification records^18^ from nearby cores in the same region. The yellow band represents lower concentrations of archaeol diether lipids coinciding with the low precipitation of the mid-Holocene from 6.4 to 4 ka BP.

The Hongyuan peats contain a range of lipid biomarkers that are commonly observed in such settings, including bacterial and archaeal dialkyl glycerol diether (DAGE) lipids (such as archaeol and *sn*-2- and *sn*-3-hydroxyarchaeols, that are the focus of this investigation, as well as non-isoprenoidal DAGEs; Supplementary Fig. 1 and Supplementary Note 1) and glycerol dialkyl glycerol tetraethers. Previously obtained humification indices and δ^13^C values of *C. muliensis* cellulose from the same peat (but a different core, from within tens of metres) have been interpreted as proxies for peat wetness, and by extension the amount of precipitation—or more precisely the net precipitation–evaporation (P–E) balance—which is related to the ASM^17^,^18^ (Supplementary Note 2). In the Hongyuan Peat, both proxies record similar trends, indicating an interval of enhanced P–E from 11.5 to 6.4 ka BP, followed by a dramatic weakening from 6.4 to 4 ka BP, after which peat wetness increased again in the late Holocene but remained lower than that of the early Holocene (Fig. 2e,f).

**Figure 3:**
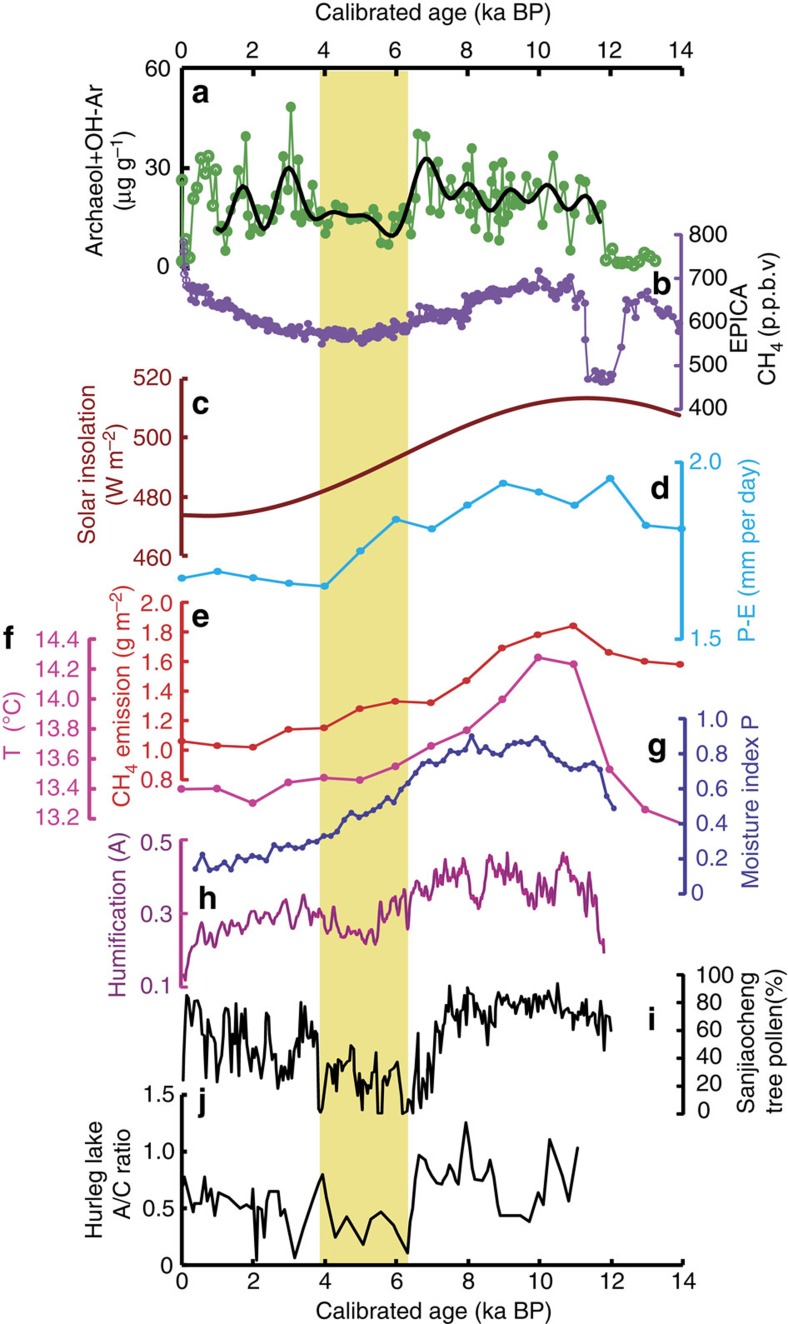
Methanogen biomass proxy and other records. (**a**) Archaeol and OH-Ar (hydroxyarchaeol) concentrations from the Tibetan peats. The black line is the low-pass filtering date showing the long-term trend. (**b**) EPICA ice core CH_4_ record^62^. (**c**) Summer solar insolation^63^. (**d**–**f**) Modelled precipitation–evaporation (P–E), CH_4_ emissions and temperature from the Tibetan Plateau (Fig. 1, derived using approaches described in ref. 2). (**g**) Moisture index based on carbonate δ^18^O in the monsoon region of China^8^. (**h**) Humification record from Hongyuan Peat^18^. (**i**) Tree pollen record from Sanjiancheng^64^. (**j**) *Artemisia/Chenopodiaceae* (A/C) ratio from Hurleg lake^65^. The yellow band represents lower methanogenesis coinciding with the low precipitation of the mid-Holocene from 6.4 to 4 ka BP.

This overall temporal pattern of decreasing precipitation during the Holocene resembles other Holocene precipitation records across the AM-influenced region, that is, Dongge Cave, southern China^5^ and Indian summer monsoon records^19^. Specifically, the Holocene optimum with maximum effective precipitation for the monsoon region occurs from ca. 10.5 to 6.5 ka BP^8^. The long-term decrease in precipitation after 6 ka BP suggests that similar monsoon precipitation trends dominate over large areas. Indeed, modelled P–E for the region using the coupled ocean-atmosphere Hadley Centre climate model (HadCM3) also indicates a decreased effective precipitation through the Holocene (Fig. 3d).

However, the Hongyuan Peat δ^13^C values^17^ and humification records^18^ do not suggest only a monotonic decrease in Tibetan Plateau precipitation but instead document a long-term drying overprinted by a pronounced dry interval from 6 to 4 ka. Other lines of evidence for this dry mid-Holocene interval include tree pollen from Sanjiaocheng (Figs 1c and 3i) and reconstructed *Artemisia*/*Chenopodiaceae* ratios from Hurleg lake (Figs 1c and 3j), also located at the edge of the current AM region. This temporal pattern has been confirmed by other geochemical proxy records (sediment carbonate content, ostracod δ^18^O values and trace elemental ratios) in the same region^20^.

### Methanogenesis associated with ASM

**Figure 4:**
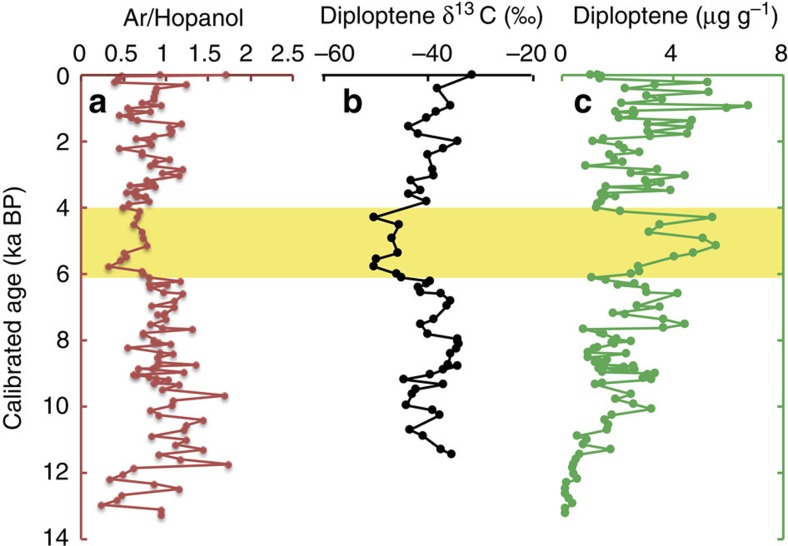
Aerobic bacterial biomass proxy records. (**a**) The ratio of archaeol to hopanol (Ar/Hopanol). (**b**,**c**) Diploptene carbon isotopes and diploptene concentrations. The yellow band represents the mid-Holocene methanotrophy maximum.

In the Hongyuan Peat, archaeol concentrations vary between 1.5 and 35 μg g^−1^ during the Holocene (Fig. 2a), being generally high during the early Holocene but low during the interval from ca. 6.4 to 4 ka BP. After 4 ka BP, they are variable but generally high, with peaks occurring at depths of 75–89 cm (1.5–1.8 ka BP) and 139–159 cm (2.8–3.3 ka BP). Although archaeol is relatively recalcitrant^21^,^22^, even being found in 50 million-year-old Eocene sediments^23^, it can be degraded in some settings^24^. If that was the case, we would expect concentrations to decrease downcore and that is not observed. Nonetheless, it is useful to constrain this by normalizing archaeol concentrations to a compound with a similar, that is hydroxylated, chemical functionality. The ratios of archaeol to hopanol (Ar/Hopanol) yield trends similar to those obtained solely from archaeol concentrations (Figs 2a,b and 4a). Hopanols in peat sequences have been argued to represent aerobic bacterial biomass, with lower concentrations being associated with higher water tables^25^. Therefore, the minimum in Ar/Hopanol ratios from ca. 6.4 to 4 ka is evidence for a minimum in the size of anaerobic, relative to aerobic, microbial populations, as well as evidence against downcore degradation of archaeol.

Hydroxyarchaeol also occurs in the Hongyuan peats as both the *sn*-2 and *sn*-3 isomers, but its concentration is much lower than that of archaeol. The presence of hydroxyarchaeol in these peats is consistent with microbiological characterizations of the nearby Zoige peat: both 16S rRNA and mcrA gene homology analyses show that *Methanosarcinales* and *Methanomicrobiales* constitute the majority of methanogens^26^, and hydroxyarchaeol is particularly abundant in species of the former^27^,^28^. The depth profiles of archaeaol and hydroxyarchaeol concentrations are similar (Fig. 2a,c,d). However, a similarity of archaeol and hydroxyarchaeol depth profiles has not been observed in other settings and is unexpected. Archaeol is relatively recalcitrant and well preserved during diagenesis^22^, whereas hydroxyarchaeol appears to be poorly preserved^9^. Consequently, the former is typically interpreted as reflecting a mixture of living but mostly fossil biomass and the latter interpreted as predominantly deriving from living biomass.

This could indicate that both lipids reflect downcore changes in living methanogen biomass, which would be problematic for our interpretation of archaeol concentrations as indicative of past changes in methane cycling. We argue that this is unlikely because previous studies have consistently shown that the majority of methanogenesis occurs between 20 and 50 cm in peats^29^,^30^,^31^. This is related to the interaction of substrate quality and quantity, the size of the methanogen community, temperature and redox conditions as dictated by water-table level. Specifically, methanogenesis predominantly occurs in the shallowest anoxic horizons where plant productivity provides abundant and labile substrates as root exudates^32^,^33^. In deeper horizons, the organic matter is older and recalcitrant, dominated by plant biomolecules and with concentrations of low-molecular-weight reactive substrates being very low^34^,^35^, causing substrate limitation of methane production^36^. Therefore, although methanogenesis is likely occurring throughout the Hongyuan peat profile, most of it is occurring in shallow peats. By extension, downcore archaeal diether lipid profiles record a history of this shallow methanogenesis intensity^9^,^11^,^30^,^36^, and we suggest that the similarity in archaeol and hydroxyarchaeol profiles in the Hongyuan peat is because of remarkable preservation of the latter in this relatively cold setting.

Thus, features in the shallow Hongyuan peat, such as peaks in archaeol concentration in the upper 70 cm (latest Holocene), must be interpreted cautiously. Instead, we focus our interpretation on the pronounced minimum in archaeal diether concentrations (and Ar/hopanol ratios) that occurs between 6.4 and 4 ka. It is very difficult to envision a scenario in which this reflects the living methanogen population, and the broad similarity between archaeal diether lipid concentrations and climate proxies is remarkable. The minima in δ^13^C values of *C. muliensis* cellulose and humification records^17^,^18^ coinciding with minima in archaeal diether concentrations and Ar/hopanol ratios suggest a link between peat wetness and depth of the water-table level and methanogen biomass (Fig. 2). We argue that intervals with high concentrations of archaeol and hydroxyarchaeol are indicative of enhanced methanogen biomass, and by extension methanogenic activity, occurring under anaerobic, that is, relatively wetter climate conditions, and *vice versa*^9^ (Fig. 2). Markedly lower concentrations of archaeal diether lipids coincide with the low P–E of the mid-Holocene from 6.4 to 4 ka BP, recorded both elsewhere on the Tibetan Plateau and more widely in the AM region (Figs 1c and 3h–j). After 4 ka BP, the methanogen biomass increases again but varies markedly, possibly at millennial scales, although these shallow records could also reflect the influence of currently living organisms. This interval corresponds to a generally weak ASM but elevated Tibetan peatland wetness^5^,^17^,^18^ (Fig. 3g,h). Overall, a close linkage between precipitation and methanogenesis is consistent with investigations of the nearby Zoige wetland of the Tibetan Plateau, where archaeal community (methanogen) abundances are 10-times lower during drought years^37^.

### Methanotrophy during the mid-Holocene

The decrease in methanogen biomass at ~6.4 ka likely reflects decreased CH_4_ production and provides direct evidence for previous hypotheses that this caused lower CH_4_ emissions. However, neither those models nor our archeal ether lipid data account for possible changes in methanotrophy. To examine this, we have also determined the carbon isotopic composition of diploptene (Fig. 4b), a relatively taxonomically widespread bacterial hopanoid that is produced by, but not exclusive to, methanotrophs^10^,^38^. The concentration of diploptene varies through the Hongyuan peat profile but does exhibit a maximum from 6.4 to 4 ka (Fig. 4c). This could be evidence for a larger aerobic bacterial population during a relatively dry climatic interval; however, given diploptene’s diverse sources, the increased concentration is not unambiguously indicative of a larger methanotroph population. Such insight can be provided by the carbon isotopic composition of diploptene^10^, which effectively records the mass balance mixing of different (putatively heterotrophic and methanotrophic) sources. Diploptene δ^13^C values vary dramatically, from −31.6‰ to −50.3‰ (Fig. 4b), with the lowest values (between −42.0‰ and −50.3‰) occurring from ~6.4 to 4 ka (Fig. 4b). This depletion is striking and yet to be observed in modern peats, where carbon isotopic compositions range from −29‰ to −34‰ (refs 10, 37, 38). Previous workers^10^ have argued that diploptene δ^13^C values as low as −34‰ could be evidence of a methanotroph contribution because the purely heterotrophic end member value apparently ranges from −20 to −30‰ (ref. 39). Therefore, the diploptene δ^13^C values below −40‰ in the 6.4- to 4-ka interval indicate a relatively large methanotroph population.

Previous work suggests that lower diploptene δ^13^C values, that is, elevated methanotrophy, are associated with greater methane fluxes^10^. We observe the opposite here, with the mid-Holocene minimum in methanogen biomarker concentrations during a relatively dry interval corresponding to a maximum in the methanotroph isotopic signature. Thus, we interpret the minimum in diploptene δ^13^C values as a change in methane flux pathways at a time when overall methane production was lower. In many wetlands, CH_4_ is transported directly from deep soil (deeper than 20 cm) to the atmosphere by the passive CH_4_ flow induced by the aerenchyma of vascular plants^40^. Indeed, the dominant sedge species of the Tibetan Plateau have been shown to mediate CH_4_ transport^41^,^42^. Under such conditions, methanotrophy is limited and diploptene δ^13^C values are expected to reflect a heterotrophic signature. Crucially, however, even in settings characterized by efficient CH_4_ oxidation, diploptene δ^13^C values are rarely lower than −40‰ (ref. 10). Therefore, we suggest that the mid-Holocene methanotroph maximum in the Hongyuan peat reflects particularly efficient oxidation of methane.

The causes of increased methane oxidation are unclear but are likely related to concomitant changes in either water-table level and/or vegetation. As discussed above, the inferred decrease in P–E in the Hongyuan Peat was likely associated with a deeper water-table level. This would have been associated with deeper oxygen penetration and methane oxidation, possibly to depths below the rooted zone. If so, methane flux across the anoxic–oxic interface could have been predominantly diffusive, rather than advective through plant vascular systems, giving rise to a greater efficiency of methane oxidation^43^,^44^. Alternatively or additionally, a plant community with a shallower root system could have the same impact. We have no direct evidence for a major change in peat type during the mid-Holocene; however, the percentage of wetland pollen is low during the period from 8 to 3 ka BP (ref. 45). Similarly, C_23_/C_31_ *n*-alkane ratios and *P*_aq_ ratios (Supplementary Note 3 and Supplementary Fig. 2) do vary in the peat profile, with a prolonged minimum in both occurring during the mid-Holocene. This is consistent with the expected shift towards peat vegetation assemblages typical of drier settings^46^,^47^,^48^,^49^. However, it also suggests the development of longer and thicker sedge roots rather than the shallower roots associated with, for example, mosses, that would cause increased methane oxidation efficiency^40^,^41^,^42^. It is possible that root-facilitated transport of O_2_ into the soil fostered increased CH_4_ oxidation in the rhizosphere^50^. Therefore, we propose that the unexpectedly high methane oxidation efficiency arose from either a diffusive flux regime or rhizosphere oxidation associated with deeper roots, both of which are consistent with drier climatic conditions.

## Discussion

Although peatland emissions are strongly modulated by a range of environmental and vegetation controls, the close correlation of methanogen biomass with bog wetness validates hypotheses that the mid-Holocene mimimum in atmospheric methane was associated, at least in this region, with decreased peatland wetness and methanogenesis. Our methanotroph signatures further suggest that this lower methane production was associated with more efficient methane oxidation. Therefore, we conclude that there is a causal link between the ASM system and methane emissions from Asian wetlands through the Holocene. However, we note that the link is not simple: archaeol concentrations in the early and late Holocene are similar, whereas P–E is apparently higher in the early Holocene. Therefore, precipitation intensity is probably not be the only control on methanogenesis.

To explore this further, we have compared our results to modelled Holocene temperature, precipitation and methane emissions from the Tibetan Plateau (Fig. 3d–f) and globally^2^. In these simulations, emissions from low-latitude northern hemisphere monsoon regions decrease in response to an orbitally induced decrease in summer insolation in the tropics (Fig. 3c), southwards migration of the ITCZ and an associated reduction in summer precipitation, in agreement with previous arguments^51^. However, although the simulations indicate markedly lower methane emissions at 4 ka relative to 10 ka across SE Asia (Fig. 1b), they indicate only a modest decrease over the Tibetan Plateau (Figs 1a and 3). This could be indicative of a sensitive response at marginal AM settings that is recorded by sediments but not by our simulations.

These model results allow some extension of our findings to wider geographical regions. CH_4_ emissions in tropical soils, including but not limited to peat, are controlled by the water-table depth^52^, and this has been the basis for arguing that the atmospheric CH_4_ temporal trends should track that of monsoon precipitation^53^. During the mid to late Holocene, the generally declining monsoon precipitation in China is consistent with that of orbital-scale CH_4_ variations, which are primarily controlled by the strength of tropical monsoons^1^ as well as with model predictions^2^ and our Tibetan wetland biomarker results. Thus, although wetlands in the Tibetan Plateau are not the largest sources of atmospheric CH_4_, the decreasing AM precipitation in China during the middle Holocene could be representative of the tropical Northern Hemisphere’s influence.

However, our modelled methane emissions for the Tibetan Plateau exhibit a decrease from 11 to 2 ka, which is largely driven by modelled cooling and decreased P–E (Fig. 3d–f); however, the humification indices and methanogen biomarker concentrations suggest that the minimum in P–E and methanogenesis occurred from 6.4 to 4 ka. It is possible that the methanogen biomarker record is biased in shallow peat by the presence of a living, modern methanogenic community^9^, but that cannot fully explain the discrepancy because it is also apparent in the precipitation records (Fig. 3).

We suggest that orbitally forced climate simulations are capturing the widescale processes related to the AM that dictate precipitation and methanogenesis in soils across China (Fig. 1a,b), but that in the Tibetan Plateau secondary processes impose additional controls. In particular, the differences between the simulations and proxy records primarily arise from the 6- to 4-ka dry interval. This pronounced drying during the mid-Holocene is not restricted to Asia and has been inferred from lake and vegetation records in regions of Central America^54^, Africa^55^ and North and South America^56^. Thus, the more complex record in the Hongyuan Peat and elsewhere could indicate that areas at the margin of the monsoonal influence (that is, in the case of Hongyuan, being perched on the northern margin of the summer monsoon regime) are more vulnerable to ITCZ location.

The Hongyuan Peat, therefore, appears to record a combination of local, regional and global forcings, and we have interrogated these by comparing modelling results with local proxy records. First, these suggest that the Tibetan Plateau became less methanogenic during the mid-Holocene, presumably because of orbital forcing. This effect appears to have been even stronger elsewhere in East China (Fig. 1b). Second, this minimum in methane production was, at least in the Hongyuan peat, associated with a non-intuitive increase in methanotrophy that we attribute to more efficient methane oxidation (under a diffusive rather than aerenchyma-facilitated advective transport regime). Third, a weakened ASM, especially in marginal regions and at the high elevation of the Tibetan Plateau, apparently brought about a mid-Holocene minimum in CH_4_ emission from about 6 to 4 ka. Climate impacts on wetland extent and methanogenesis were likely not limited to the Tibetan Plateau, especially given the widespread, orbitally paced decrease in monsoon intensity through the Holocene^57^. Both northern and tropical peatland expansions slowed at ~5 ka and has been attributed to neoglacial cooling in high northern latitudes and weakening monsoons in low latitudes^4^. Crucially, evidence for a mid-Holocene dry interval is also widespread^54^,^55^,^56^. Therefore, the Tibetan peat data provide evidence of how monsoon-driven hydrological conditions could have more widely influenced CH_4_ emissions during the Holocene. We propose that CH_4_ emissions, at least in East Asia, were indeed controlled by interactions of large-scale atmospheric circulations, but modulated by regional factors, and that future work should explore the regional variation of these responses.

## Methods

### Site description

The Zoige-Hongyuan Peat is located on the eastern edge of the Tibetan Plateau and is the largest peatland in China^58^. The average elevation of the peatland is 3,400 m a.s.l. and the Hongyuan Peat sampling site is 2 km southeast of Hongyuan County at 32°46′N, 102°31′E, with an altitude of 3,507 m (Fig. 1c). The Hongyuan Peat covers an area of ~4,500 km^2^. The area is characterized by cold and wet climate and a long frost period. The annual mean temperature is ~1 °C, the January mean temperature is approximately −10.9 °C and the July mean temperature is ~11 °C. The annual mean precipitation is ~700 mm. The climate of this area is mainly controlled by the AM systems (Fig. 1c). The continuous Hongyuan Peat core was recovered using a Russian peat corer. The core is 754 cm long and consists of 584 cm of brown to dark-brown acid peat containing a large amount of undegraded plant residue, underlain by 6 cm of dark-brown mud and then 64 cm of dark-brown peat. Below 654 cm depth, the sediment is greyish-green to dark-brown mud, representing lacustrine depositional conditions. The core was subsampled in the laboratory at 1-cm intervals to conduct biomarker measurements.

### Lipid biomarker analysis

Freeze-dried, homogenized samples were extracted by sonication with a sequence of increasingly polar solvents, 3 × with dichloromethane (DCM), 3 × with DCM/methanol (1:1 v/v) and 2 × with methanol. The total lipid extracts were separated into neutral, free fatty acids and phospholipid fractions using an aminopropylsilyl bond elute column (Biotage), cleaned before use with 18 ml successive rinses of MeOH followed by 2:1 DCM: 2-propanol. At least 12 ml each of 2:1 (v/v) DCM:isopropanol, 2% glacial acetic acid in ethyl ether and MeOH were used to elute the neutral, free fatty acid and phospholipid fractions, respectively. Neutral fractions were separated further using a column packed with (activated) alumina by elution with hexane/DCM (9:1 v/v; 9 ml; apolar fraction) and DCM/methanol (1:1 v/v; 9 ml; polar fraction), respectively. An aliquot of the polar fraction was silylated with pyridine and N,O-bis(trimethylsilyl)trifluoroacetamide (BSTFA) at 70 °C for 1 h and dissolved in hexane before analysis using gas chromatography–mass spectrometry (GC/MS). Fractions were analysed with GC/MS using a ThermoQuest Finnigan Trace GC and MS instrument equipped with a non-polar silica CP-Sil-5–CB column (50 m × 0.32 mm with a 0.12-μm film thickness) using the following temperature programmes: 70–130 °C at 20 °C min^−1^, ramp to 300 °C at 4 °C min^−1^ and held at 300 °C for 20 min. The ionization potential was 70 eV, with the scanning range m/z 50–650. The peak areas for hydroxyarchaeol and diploptene were calculated using mass chromatograms (m/z 143 and 191, respectively) and converted to concentrations using response factors.

### Carbon isotopic analysis

Diploptene δ^13^C values were determined using gas chromatography–isotope ratio mass spectrometry (GC–IRMS) with a ThermoFisher Delta V. A fused silica capillary column (60 m × 0.32 mm) coated with CP-Sil-5 (film thickness 0.10 μm) was used with the same GC temperature programme as above. The δ^13^C values are reported relative to the Vienna Pee Dee Belemnite (VPDB) standard, and the analytical error, determined by using co-injected standards, is ±0.5‰.

### AMS ^14^C dating and chronology

Sample pretreatment, AMS-target preparation and AMS measurement were all conducted at the State Key Laboratory of Loess and Quaternary Geology (SKLLQG).The pretreatment of samples for ^14^C dating was performed using the method of ref. 59: plant fragments with a size ranging between 90 and 300 μm were isolated from peat by wet sieving, and then subjected to an acid–alkali–acid (HCl–NaOH–HCl) treatment^59^. AMS targets were prepared from the pretreated samples, which were then placed with CuO powder into 9-mm quartz tubes, evacuated to <10^−5 ^torr and then combusted. The CO_2_ was converted catalytically to graphite using Zn (Zn powder with added Fe powder as a catalyst)^60^. Dating was calibrated using the Calib611 programme^61^. The average value of the 2σ calibrated age range is quoted as the calibrated age. The calibrated ages provide a firm chronological framework for the past 13,000 years. The results of two separate parts of the studied section are least square-fitted to establish the chronological framework (Supplementary Fig. 3 and Supplementary Table 1).

### Climate model

The Hadley Centre coupled ocean-atmosphere model, HadCM3, was used to perform equilibrium snapshot simulations for the Holocene. The simulations are equivalent to the ‘ALL’ experiment described in ref. 2, where each time-slice simulation has changed to boundary conditions ice-sheet volume, land–sea mask, trace greenhouse gases and orbital configuration derived from palaeo data. Time slices were set up at 1-ka intervals for the Holocene and each was run for 500 years. The results presented here are climatologies from the last 30 years of each simulation. Further details can be found in ref. 2.

## Author contributions

The model work was carried out by J.S.S and P.J.V., and the AMS ^14^C age data were analysed by P.C. Y.Z., Z.L. and X.Y. performed the field work; Y.Z. conducted biomarker measurement, analysis and interpretation. R.D.P. and Y.Z. designed the project and the manuscript was written by Y.Z. and R.D.P with contributions from all authors.

## Additional information

**How to cite this article:** Zheng, Y. *et al.* Holocene variations in peatland methane cycling associated with the Asian summer monsoon system. *Nat. Commun.* 5:4631 doi: 10.1038/ncomms5631 (2014).

## Supplementary Material

Supplementary Information Supplementary Figures 1-3, Supplementary Table 1, Supplementary Notes 1-3 and Supplementary References
