# Supplementary material for: Holocene variations in peatland methane cycling associated with the Asian summer monsoon system
Source: Nat Commun. 2014 Aug 19;5:4631. doi: 10.1038/ncomms5631 (PMC4143914; doi:10.1038/ncomms5631)
Supplement: Supplementary Information — Supplementary Figures 1-3, Supplementary Table 1, Supplementary Notes 1-3 and Supplementary References [file ncomms5631-s1.pdf]

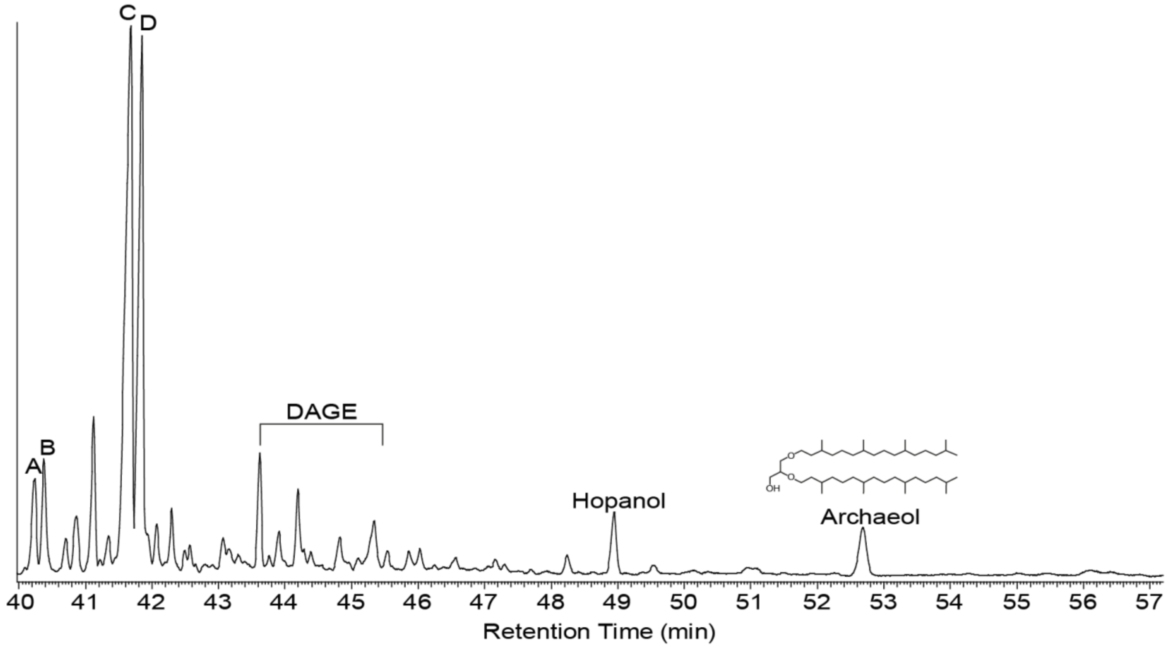

Supplementary Figure 1. Partial total ion current (TIC) chromatogram of polar fraction (as TMS derivatives). The TIC shows relative abundances of archaeol, bacterial dialkyl glycerol diethers (DAGEs) and the main sterols at the depth of 49cm (950 cal a BP) in the Hongyuan Peat from the Tibetan Plateau. A, 24-methylcholest-5-enol; B, 24-methylcholestanol; C, 24-ethylcholest-5-en-ol; D, 24-ethylcholestanol. The *sn*-2- and *sn*-3-hydroxyarchaeol compounds elute at 56 to 57min but are not apparent in the TIC.

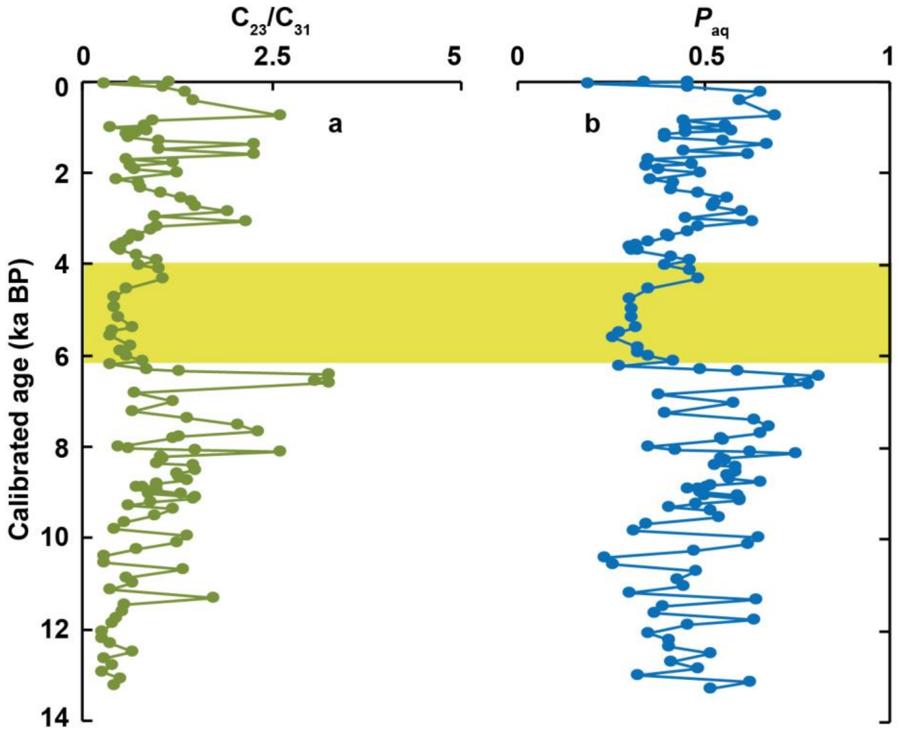

Supplementary Figure 2. The ratio of C_{23} to C_{31} *n*-alkane (C_{23}/C_{31}) and P_{aq} (P_{aq}= (C_{23}+C_{25})/(C_{23}+C_{25}+C_{29}+C_{31}) values at Hongyuan Peat. The yellow band denotes the lowest C_{23}/C_{31} and P_{aq} values which suggest that the drier conditions during the mid-Holocene resulted in a deeper water table level and a corresponding vegetation change.

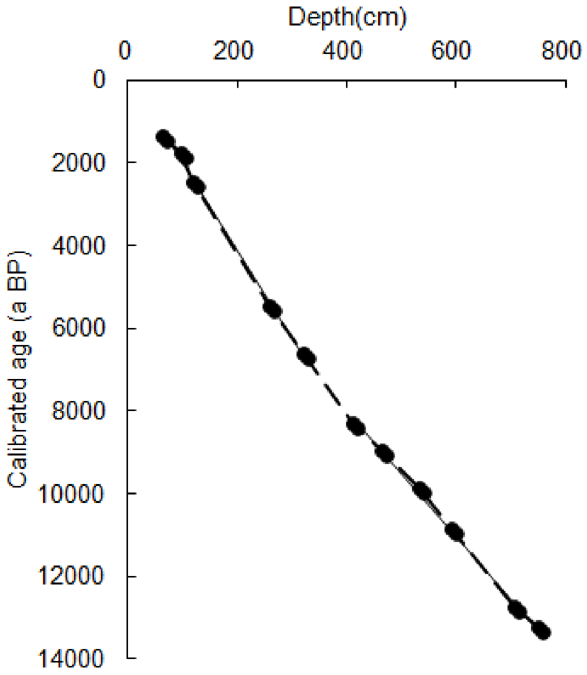

Supplementary Figure 3. Calibrated radiocarbon ages plotted against peat depth. Ages between measured horizons are obtained by least square fit.

Supplementary Table 1. Results of ^{14}C AMS dating from Hongyuan Peats of the Tibetan Plateau.

Depth(cm)	Date material	^{14}C age		Calibrated age (cal a BP)		
		(a BP)	Uncertainty(a)	Lower	Upper	Average*
63	Plant fragments	1459	32	1302	1398	1350
96	Plant fragments	1825	33	1634	1864	1749
118	Plant fragments	2350	39	2213	2675	2444
258	Plant fragments	4764	39	5330	5589	5460
322	Plant fragments	5836	39	6509	6742	6626
412	Plant fragments	7531	44	8207	8413	8310
463	Plant fragments	8074	49	8774	9128	8951
531	Plant fragments	8778	48	9561	10119	9840
592	Plant fragments	9484	48	10585	11070	10828
705	Plant fragments	10828	86	12575	12914	12745
750	Plant fragments	11322	66	13093	13345	13219

*Average age is obtained by the average values of lower calibrated age and upper calibrated age.

Supplementary Note 1

Archaeal diether distributions

The Hongyuan peats contain a range of lipid biomarkers that are commonly observed in such settings, including a range of *n*-alkanes, *n*-alkanols, *n*-alkanoic acids, stenols/stanols, triterpenoids, hopanoids, bacterial and archaeal dialkyl glycerol diether (DAGE) lipids (such as archaeol and hydroxyarchaeol, as well as non-isoprenoidal bacterial DAGEs; Supplementary Fig. 1) and glycerol dialkyl glycerol tetraethers (GDGTs). The archaeal and bacterial DAGEs, including archaeol and hydroxyarchaeol (*sn*-2 and *sn*-3 isomers), are present in all samples. Archaeol concentrations vary between 1.2 and 2.3 μg g^{-1} in the underlying lacustrine interval (Supplementary Fig. 1). The bacterial DAGEs bear primarily C_{15}-C_{17} alkyl components, including both straight-chain and branched structures.

Supplementary Note 2

$\delta^{13}C$ values of *C. muliensis* cellulose and humification

The $\delta^{13}C$ values of cellulose from the modern dominant plant (*Carex muliensis*) in the Hongyuan Peat were determined by ref.1. The $\delta^{13}C$ values of these major peat-forming plants are higher when relative humidity and/or temperature is lower[2], although the former is typically considered to be the major control[3]. Therefore, a decrease in relative humidity or increase in temperature results in an increase in the $\delta^{13}C$ value[2], and vice versa. The moisture at the bog surface in the Hongyuan region primarily depends on rainfall caused by the Asian summer monsoon[1], such that variations in the $\delta^{13}C$ value of Hongyuan peat plant remains are considered to be proxies for the monsoon[1].

Humification has a positive correlation to both temperature and soil wetness when the temperature is lower than 30°C and the soil wetness is lower than 80% (ref.4), such that higher humification indices indicate a wetter-warmer climate[5].

Supplementary Note 3

Biomarker-based evaluation of vegetation change

In order to qualitatively evaluate changes in peat-forming vegetation, we examined the distribution of *n*-alkanes[6,7,8]. In European ombrotrophic bogs, medium chain length (C_{23}-C_{25}) *n*-alkanes are produced mainly by *Sphagnum* and submerged vascular macrophytes, whereas the longer chain components (C_{29}-C_{31}) are produced mainly by terrestrial vascular plants as epicuticular leaf waxes[6,7,9]. The ratio of the C_{23}/C_{31} *n*-alkanes and the P_{aq} values of *n*-alkanes (Defined in Supplementary Fig. 2) are lowest during the mid-Holocene, and higher during the early Holocene and late Holocene (Supplementary Fig.2). This suggests that the drier conditions during the mid-Holocene resulted in a deeper water table level and a corresponding vegetation change, a co-variation that has been observed in a variety of other peat deposits[6,10].

Supplementary References

1. Hong, Y.T. *et al.* Correlation between Indian Ocean summer monsoon and North Atlantic climate during the Holocene. *Earth Planet. Sci. Lett.* **211**, 371–380 (2003).

2. Edwards, T.W.D. Graf, W., Trimborn, P., Stichler, W., Lipp, J.& Payer, H.D. **δ^{13}C** response surface resolves humidity and temperature signals in trees, *Geochim. Cosmochim. Ac.* **64**, 161–167 (2000).

3. Schleser, G.H. *Parameters determining carbon isotope ratios in plants* (Eds Frenzel B., Stauffer, B. &Weiss, M.M.) 71–96 (Paläoklimaforschung 15, Strasbourg, 1995).

4. Chai X. *Peatland* (in Chinese) (Beijing: Geological Publishing House, 1990).

5. Yu, X. F., Zhou, W. J., Liu, Z. & Kang, Z. H. Different patterns of changes in the Asian summer and winter monsoons on the eastern Tibetan Plateau during the Holocene. *Holocene* **21**, 1031–1036 (2011).

6. Nott, C.J. *et al*. *n*-Alkane distributions in ombrotrophic mires as indicators of vegetation change related to climatic variation. *Org. Geochem.* **31**, 231–235 (2000).

7. Pancost, R.D., Baas, M., van Geel, B.& Sinninghe Damste´, J.S. Biomarkers as proxies for plant inputs to peats: an example from a sub-boreal ombrotrophic bog. *Org. Geochem*. **33**, 675–690 (2002).

8. Bingham, E.M. *et al*. Conservative composition of *n*-alkane biomarkers in *Sphagnum* species: implications for palaeoclimate reconstruction in ombrotrophic peat bogs. *Org. Geochem*. **41**, 214–220 (2010).

9. Ficken, K.J., Li, B., Swain, D.L. & Eglinton, G. An *n*-alkane proxy for the sedimentary input of submerged/floating freshwater aquatic macrophytes. *Org. Geochem*. **31**, 745–749 (2000).

10. McClymont, E.L. *et al*. Pyrolysis GC/MS as a rapid screening tool for determination of peat-forming plant composition in cores from ombrotrophic peat. *Org. Geochem*. **42**, 1420–1435 (2011).
